# Supplementary material for: Prime editing for functional repair in patient-derived disease models
Source: Nat Commun. 2020 Oct 23;11:5352. doi: 10.1038/s41467-020-19136-7 (PMC7584657; doi:10.1038/s41467-020-19136-7)
Supplement: Supplementary file 1 — Supplementary Information [file 41467_2020_19136_MOESM1_ESM.pdf]

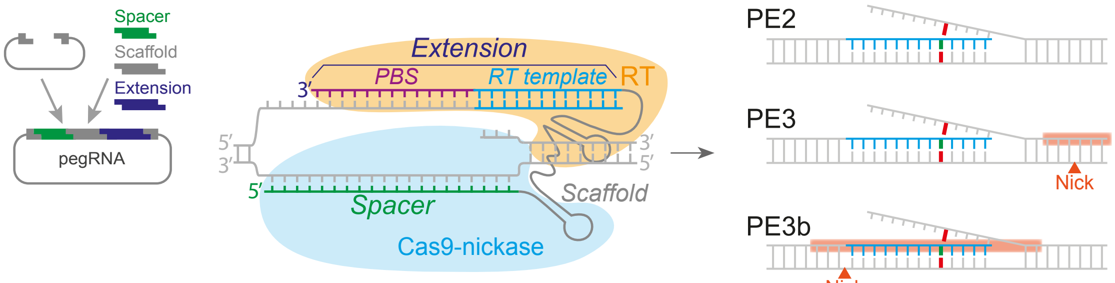

Supplementary Figure 1
Overview of the prime editing principle
Schematic representation of the pegRNA structure and the mechanism by which a pegRNA molecule binds to genomic DNA to instruct PE2 (Cas9-reverse transcriptase fusion complex) to bind to a nick a specific sequence, and synthesize a new, edited flap according to the RT template. The PE3 strategy adds a nick in the non-edited strand to enhance incorporation of the edited flap. The PE3b strategy is similar to PE3, but only nicks the opposite strand after succesful creation of an edited flap. PBS: primer binding site, RT: reverse transcriptase.

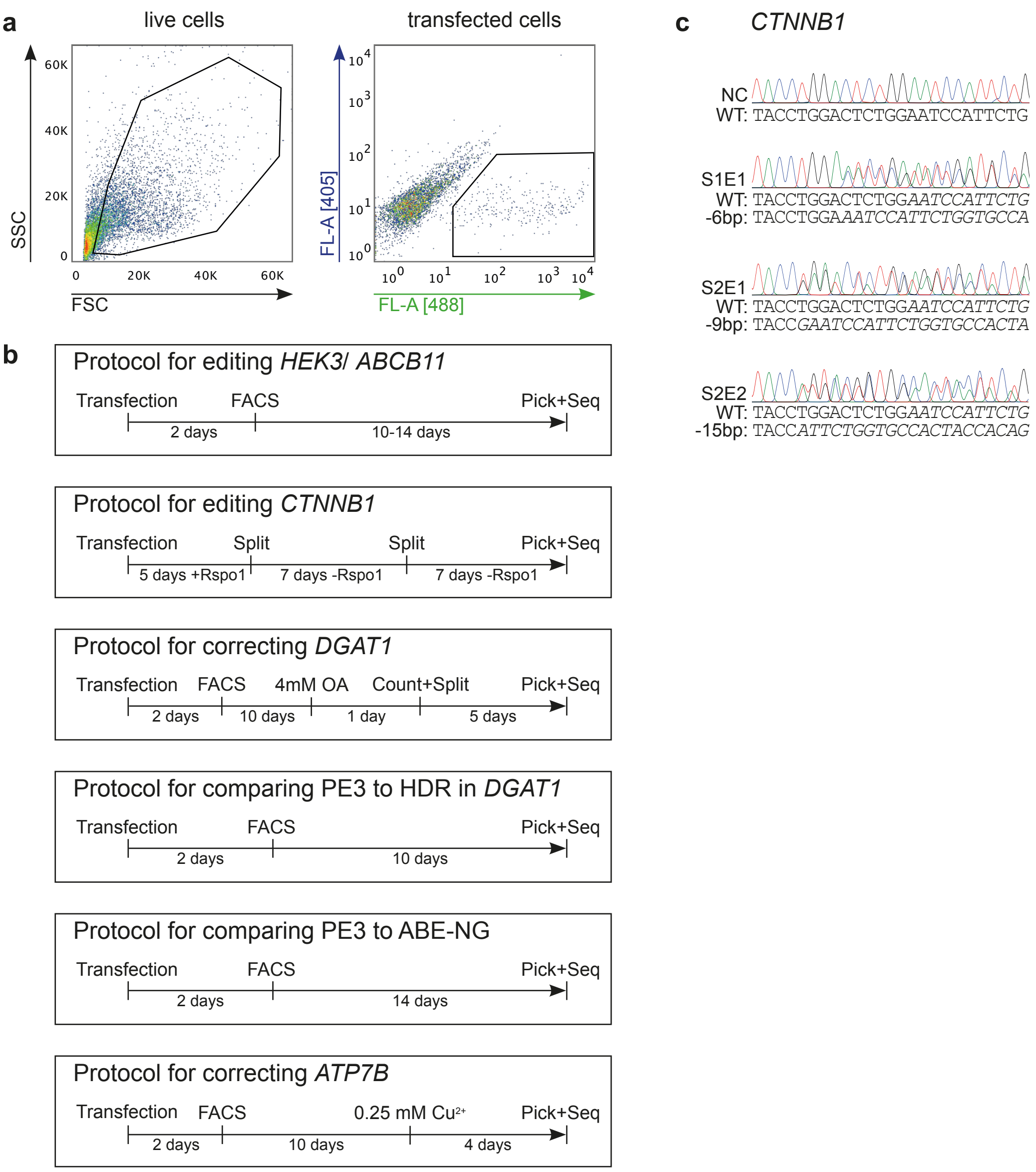

Supplementary Figure 2

Representative FACS plot example and different protocols used in this study

a, Representative FACS plot and gating strategy to sort transfected cells. Sorting of transfected cells was based on co-transfection with a GFP-reporter plasmid in all transfection experiments. **b**, Workflow and timeline for transfection of organoid cells, selection of single transfected cells, and characterization of prime editing efficiency in clonally expanded cells. Note that the workflow used for prime editing of *CTNNB1* does not contain FACS-based selection of transfected cells, but instead selects for *CTNNB1* mutants based on withdrawal of R-spondin 1 (Rspo1). **c**, Sanger validation of precise 6-bp, 9-bp, and 15-bp deletions in picked clones from *CTNNB1* pegRNA S1E1, S2E1, and S2E2, respectively. SSC: side scatter, FSC: forward scatter, OA: oleic acid.

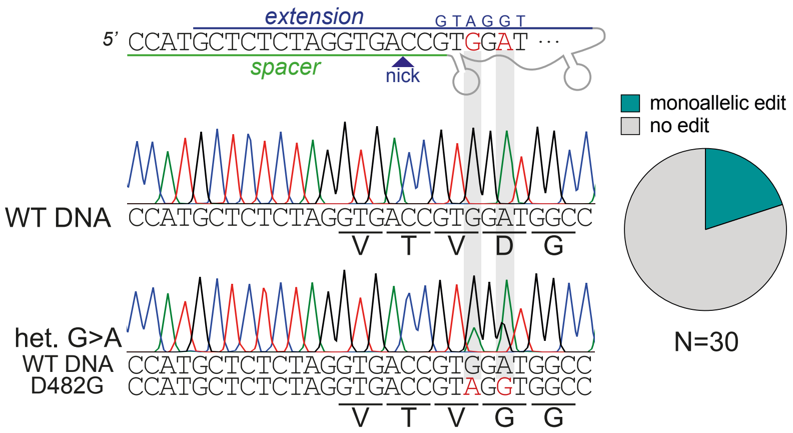

Supplementary Figure 3

Generation of *ABCB11*^{D482G} by prime editing (PE3)

Design of pegRNA and Sanger validation of generation of the pathogenic *ABCB11*^{D482G} mutation in liver organoids. Note that *ABCB11*^{D482G} is created by an A→G substitution at position 7, but the pegRNA additionally introduces a silent A→G mutation at position 5, disturbing the PAM sequence to increase efficiency.

HEK3 unwanted byproduct analysis

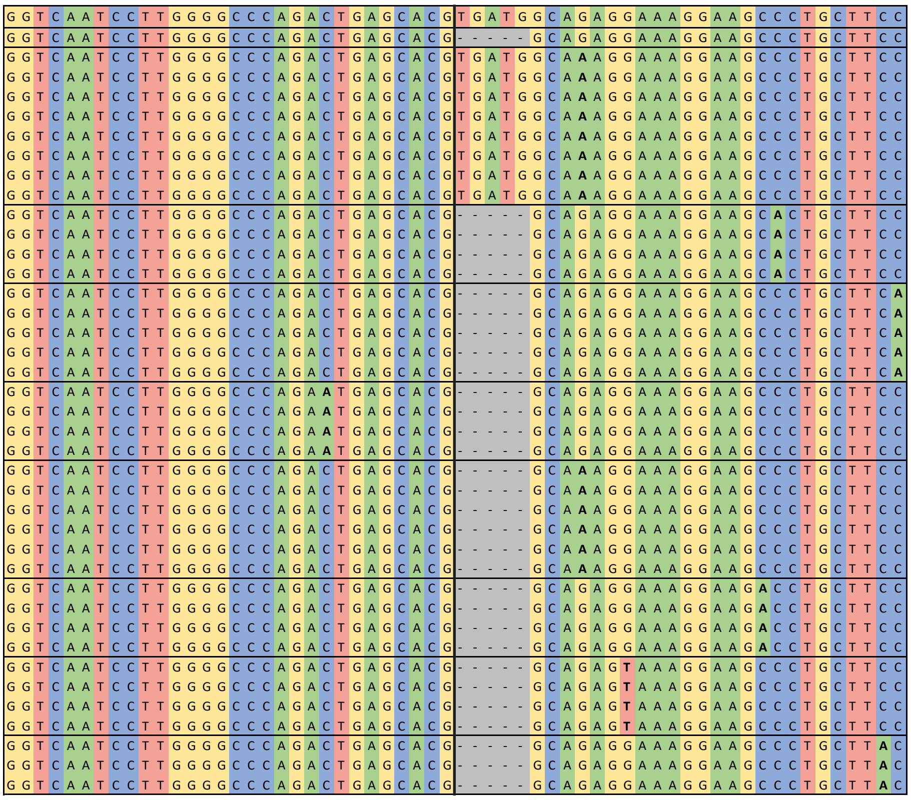

Pattern	%	Reads	Cell type
WT	Fig 1h	-	-
5bp del	Fig 1h	-	-
G>A +9	2.392	1198	intestinal
G>A +9	1.022	95	liver
G>A +9	0.898	114	intestinal
G>A +9	0.864	216	intestinal
G>A +9	0.685	217	HEK293T
G>A +9	0.682	295	HEK293T
G>A +9	0.534	311	HEK293T
G>A +9	0.271	73	liver
C>A +23	1.549	491	HEK293T
C>A +23	0.78	200	Caco-2
C>A +23	0.737	98	liver
C>A +23	0.528	132	intestinal
C>A +31	1.495	474	HEK293T
C>A +31	0.925	123	liver
C>A +31	0.74	185	intestinal
C>A +31	0.709	182	Caco-2
C>A +31	0.25	108	HEK293T
C>A -9	0.776	246	HEK293T
C>A -9	0.489	65	liver
C>A -9	0.444	114	Caco-2
C>A -9	0.412	103	intestinal
G>A +9	0.669	89	liver
G>A +9	0.446	193	HEK293T
G>A +9	0.444	111	intestinal
G>A +9	0.42	39	liver
G>A +9	0.302	176	HEK293T
G>A +9	0.28	140	intestinal
C>A +23	0.656	208	HEK293T
C>A +23	0.632	84	liver
C>A +23	0.394	101	Caco-2
C>A +23	0.328	82	intestinal
G>T +12	0.609	193	HEK293T
G>T +12	0.448	115	Caco-2
G>T +12	0.391	52	liver
G>T +12	0.364	91	intestinal
C>A +30	0.606	192	HEK293T
C>A +30	0.399	53	liver
C>A +30	0.277	71	Caco-2

CTNNB1 unwanted byproduct analysis

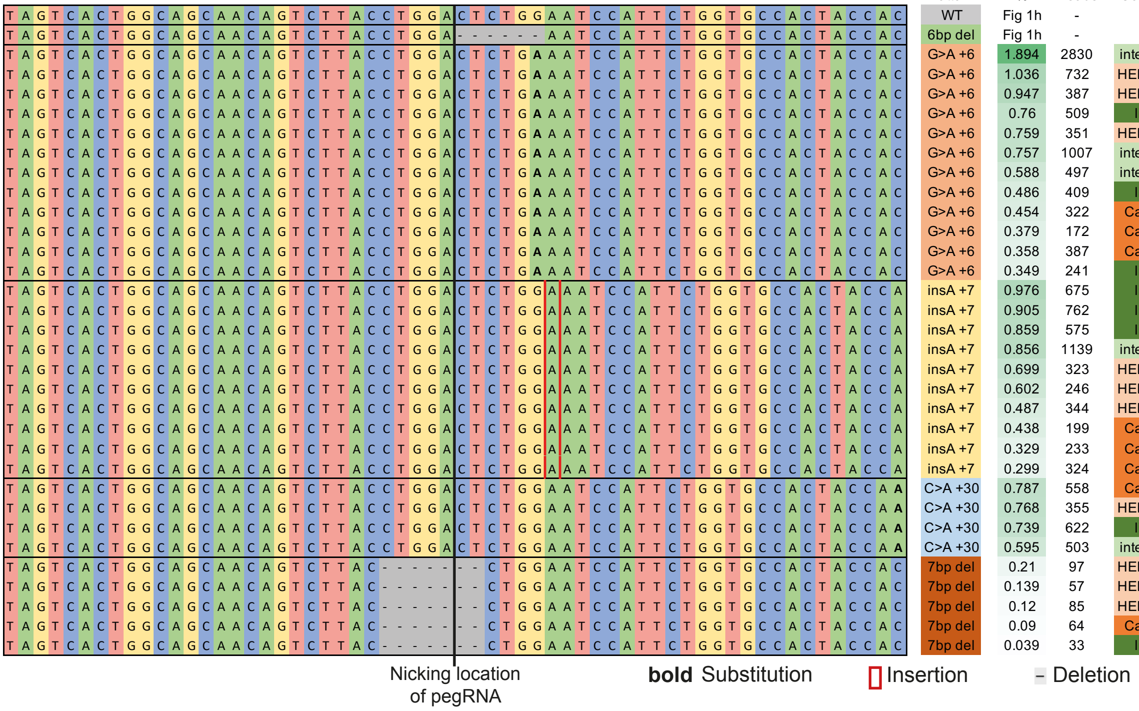

Pattern	%	Reads	Cell type
WT	Fig 1h	-	-
6bp del	Fig 1h	-	-
G>A +6	1.894	2830	intestinal
G>A +6	1.036	732	HEK293T
G>A +6	0.947	387	HEK293T
G>A +6	0.76	509	liver
G>A +6	0.759	351	HEK293T
G>A +6	0.757	1007	intestinal
G>A +6	0.588	497	intestinal
G>A +6	0.486	409	liver
G>A +6	0.454	322	Caco-2
G>A +6	0.379	172	Caco-2
G>A +6	0.358	387	Caco-2
G>A +6	0.349	241	liver
insA +7	0.976	675	liver
insA +7	0.905	762	liver
insA +7	0.859	575	liver
insA +7	0.856	1139	intestinal
insA +7	0.699	323	HEK293T
insA +7	0.602	246	HEK293T
insA +7	0.487	344	HEK293T
insA +7	0.438	199	Caco-2
insA +7	0.329	233	Caco-2
insA +7	0.299	324	Caco-2
C>A +30	0.787	558	Caco-2
C>A +30	0.768	355	HEK293T
C>A +30	0.739	622	liver
C>A +30	0.595	503	intestinal
7bp del	0.21	97	HEK293T
7bp del	0.139	57	HEK293T
7bp del	0.12	85	HEK293T
7bp del	0.09	64	Caco-2
7bp del	0.039	33	liver

Supplementary Figure 4

Analysis of editing byproducts after prime editing in HEK293T cells, Caco-2 cells, and liver and intestinal organoid cells

Allele frequency tables of the *HEK3* and *CTNNB1* locus after prime editing (PE3) to generate a 5-bp and 6-bp deletion, respectively, directly upstream from the pegRNA nicking position. For each cell type, transfection and high-throughput amplicon sequencing was performed in three replicates. Unwanted byproducts that occured at a frequency of >0.2% were grouped by pattern to vizualize occurence in each cell type. Note that common unwanted byproduct patterns occur in all cell types, but generally at a very low rate (<1%).

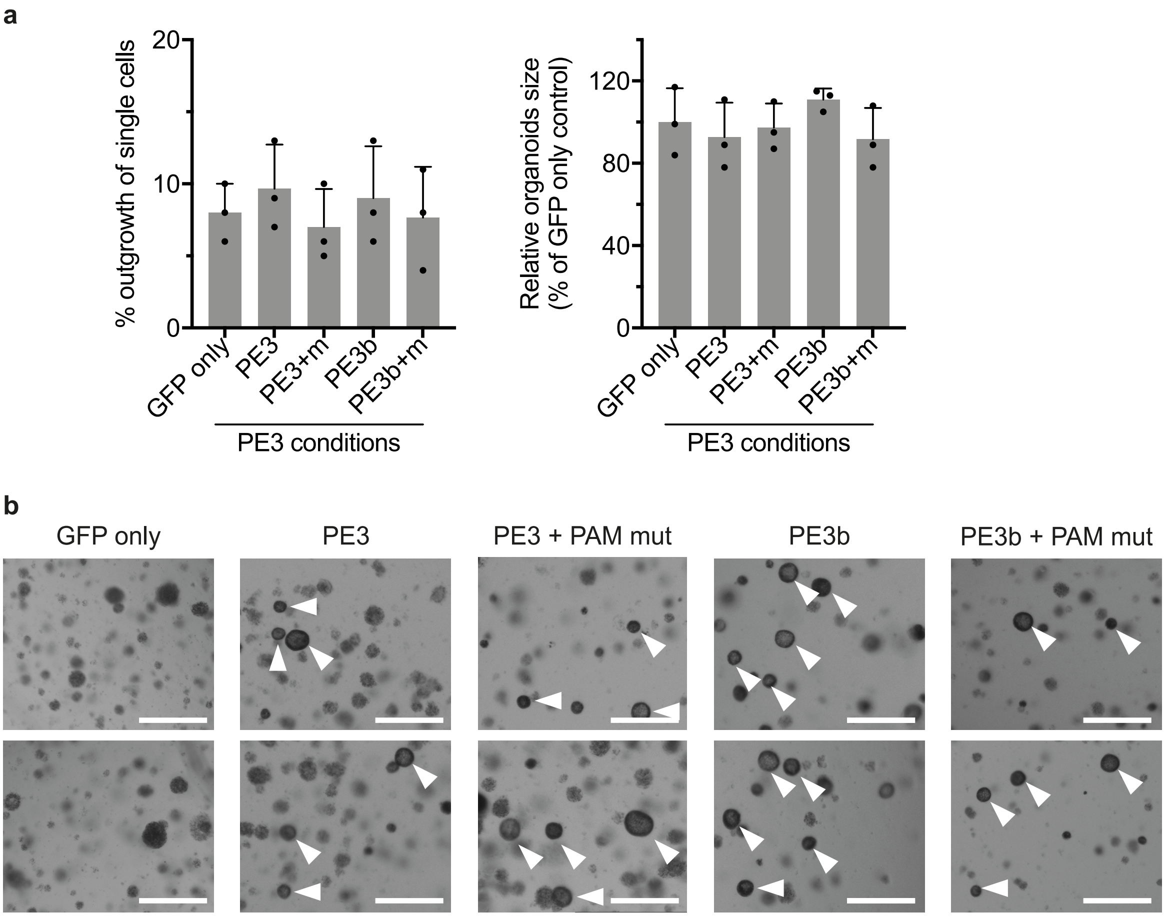

Supplementary Figure 5

Functional correction of *DGAT1*$^{\mathrm{S210del}}$ by prime editing (PE3)

a, Left panel represents outgrowth of single, FACS sorted patient-derived intestinal organoid cells after transfection with GFP-reporter plasmid only or with GFP-reporter plasmid and PE3 plasmids. Right panel represents relative organoid size at day 7 after seeding, which was used as a proxy to quantify proliferation capacity of organoid cells in each condition. Data are represented as mean values +/- S.D. of three independent experiments. **b,** Representative brightfield images of *DGAT1*$^{\mathrm{S210del}}$ patient organoids after exposure to 4 mM oleic acid for 24 hours, without (GFP only) and with prime editing (PE3/PE3b). PAM mut stands for conditions in which the PAM-site was mutated by prime editing. White arrowheads indicate surviving organoids. White scale bars are 500 µm. PE3+m: PE3 with introduction of PAM mutation. Source data are provided as a Source Data file.

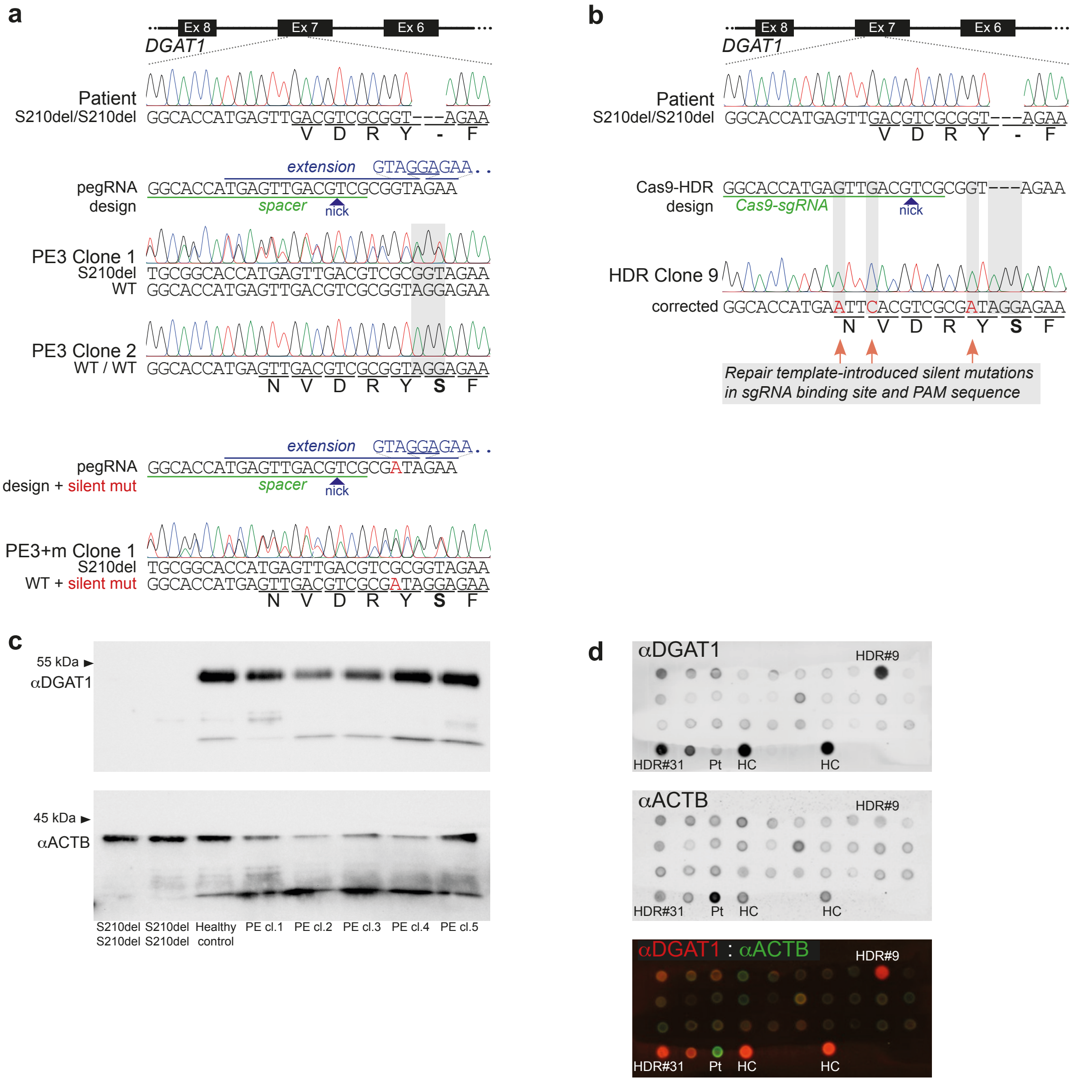

Supplementary Figure 6

Prime editing (PE3) and Cas9-mediated HDR to correct *DGAT1*^{S210del}

a, Design of the pegRNAs used in PE3 to repair the *DGAT1*^{S210del} mutation in patient-derived intestinal organoids. Sanger validation of the mutation before editing and after editing in two clonally expanded organoid lines with monoallelic and biallelic repair. Note that PE3+mutation of the PAM results in a silent mutation. **b**, Design of the sgRNA used to repair *DGAT1*^{S210del} mutation through Cas9-initiated HDR. A homology template of ±200 nucleotides flanking the DSB was used, which contained silent mutations to enhance editing. Sanger validation of the single clone with biallelic repair. **c**, Western blot validation of 5 prime edited (PE) clones. **d**, Dot blot validation of HDR corrected clones; Pt: *DGAT1*^{S210del} patient organoids; HC: healthy control organoids; HDR#9: *DGAT1*^{S210del} patient organoids after HDR correction, clone #9. Ex: exon, HDR: homology directed repair, Pt: patient, HC: healthy control.

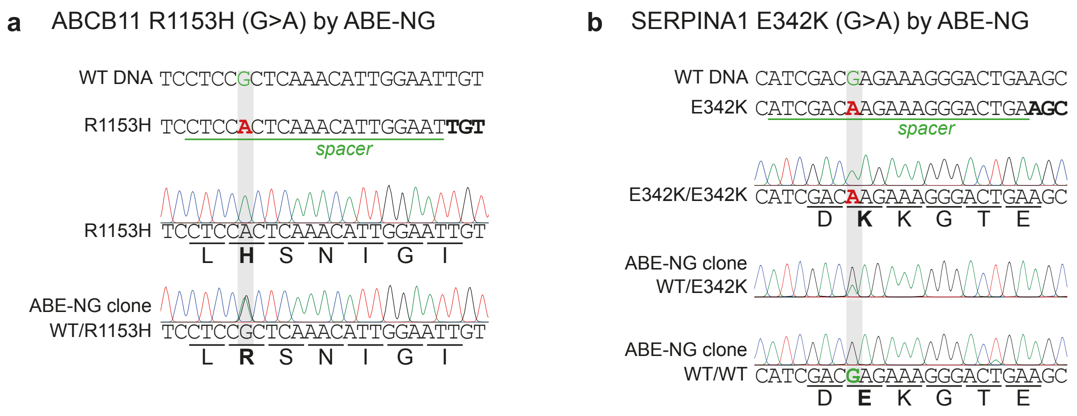

Supplementary Figure 7

Correction of *ABCB11*^{R1153H} and *SERPINA1*^{E342K} by base editing

a, Design of the sgRNA used for adenine base editing (ABE) of the *ABCB11*^{R1153H} mutation using ABEmax-NG in liver organoids derived from a patient with BSEP-deficiency. Sanger validation of monoallelic repair by ABE-NG **b,** Design of the sgRNA used for adenine base editing of the *SERPINA1*^{E342K} mutation using ABEmax-NG in liver organoids derived from a patient with alpha-1-antitrypsin deficiency. Sanger validation of monoallelic and biallelic repair by ABE-NG. The mutations in **(a)** and **(b)** were also targeted by PE3 but without success; related to Fig. 2g.

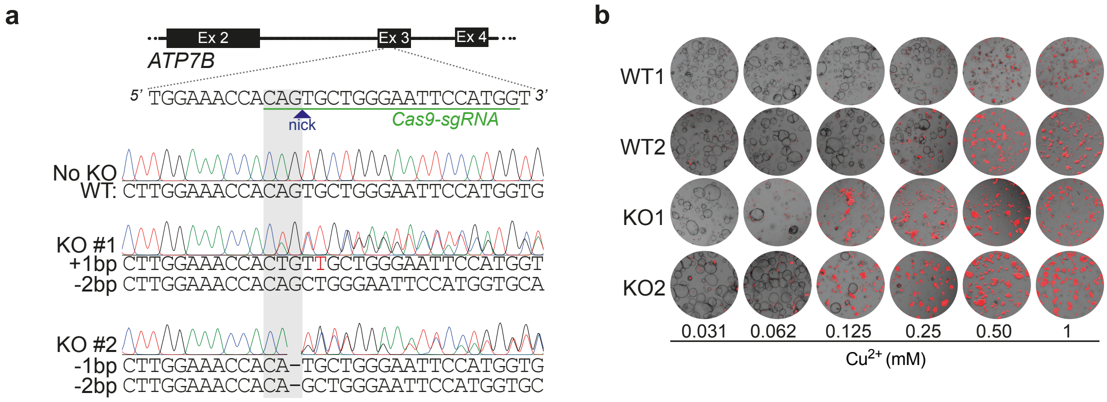

Supplementary Figure 8

Validation of a functional assay for ATP7B function

a, Design of the sgRNA used to create indels in exon 3 of *ATP7B* in liver organoids, derived from healthy controls, using a cutting Cas9. Sanger validation of two clonal *ATP7B* knock-out (KO) lines, showing heterozygous frameshift mutations. **b,** Propidium Iodide staining on wildtype and *ATP7B* knock-out liver organoids after copper exposure. Ex: exon.

a

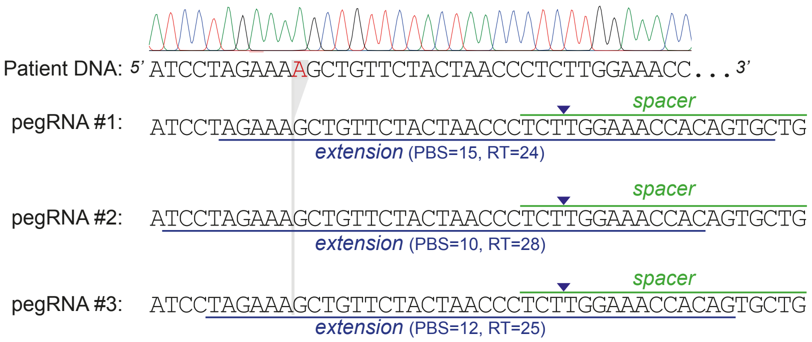

Supplementary Figure 9

PegRNA designs used for correction of *ATP7B*$^{\mathrm{S410fs}}$

Schematic overview of the different pegRNAs used in an effort to correct *ATP7B*$^{\mathrm{S410fs}}$ mutations in liver organoids derived from a Wilson disease patient. Note that the same spacer was used, but various PBS and RT-template lenghts were tested. PBS: primer binding site, RT: reverse transcriptase template.

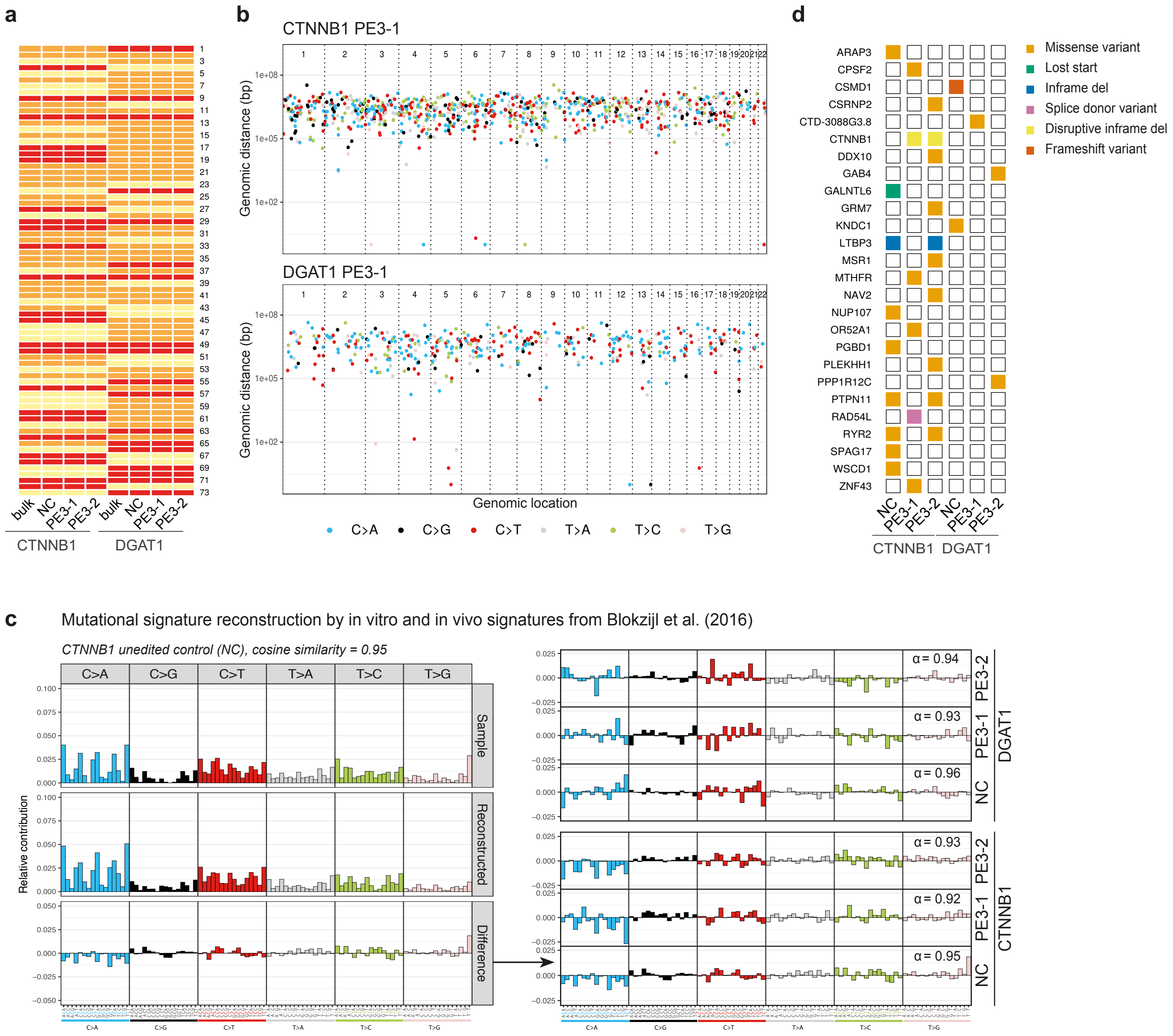

Supplementary Figure 10

Prime editing induces no genome-wide off-target effects

a, Fingerprinting of whole-genome-sequenced samples validates shared donor origin of bulk, unedited negative control (NC), and prime edited (PE3-1 and PE3-2) organoid lines. **b,** Rainfall plots of prime edited clonal organoid lines. Every new mutation, relative to the background bulk line, is indicated with a dot and is ordered by genomic location on the X axis. The Y axis shows the genomic distance between each new mutation and the one prior to it (log scale). **c,** Reconstruction of the mutational signature of prime edited and unedited control samples by *in vivo*- and *in vitro*-signatures from Blokzijl et al. (2016). Cosine similarity (α) indicates how well the reconstructed signature resembles the sample signature (1= perfect, 0.9= very well, 0= no resemblance). **d,** Overview of new variants in tumor suppressors and oncogenes from COSMIC (Forbes et al., 2017). Note that the disruptive in-frame deletions in *CTNNB1* are the intended, correct mutations of the clonal prime edited *CTNNB1* organoid lines (PE3-1 and PE3-2). Also note that prime edited samples do not acquire more mutations in the considered genes compared to negative control (NC) samples of the same donor line.

Supplementary Table 1. Sequences of pegRNAs used in prime editing experiments. All sequences are shown in 5' to 3' orientation. pegRNAs are a concatenation of the spacer sequence, the sgRNA scaffold, and the 3' extension (contains PBS and RT template (RTT)).

pegRNA	spacer sequence	3' extension sequence	PBS length (nt)	RTT length (nt)	Figure
HEK3_del1-5	GGCCCAGACTGAGCACGTGA	TGGAGGAAGCAGGGCTTCCTTTCCTCTGCCGTGCTCAGTCTG	13	29	1b,h, S4
HEK3_26CtoG	GGCCCAGACTGAGCACGTGA	TGGAGGAACCAGGGCTTCCTTTCCTCTGCCATCACGTGCTCAGTCTG	13	34	1c
CTNNB1_S1E1_del1-6	CAACAGTCTTACCTGGACTC	TGGCACCAGAATGGATTTCCAGGTAAGAC	12	17	1d,e,g,h, S4
CTNNB1_S1E2_del3-5	CAACAGTCTTACCTGGACTC	ACCAGAATGGATTCAGTCCAGGTAAGACTGT	15	16	1d,e,g
CTNNB1_S2E1_del4-12	CTGGCAGCAACAGTCTTACC	TGGTAGTGGCACCAGAATGGTAAGACTGTTGCTGCC	15	21	1d,e,g
CTNNB1_S2E2_del4-18	CTGGCAGCAACAGTCTTACC	TGGCACCAGAATGGATTCGGTAAGACTGTTGCT	12	21	1d,e,g
ABCB11-D482G-create	CCATGCTCTCTAGGTGACCG	AATGTCATGGCCACCTACGGTCACCTAGAGAGC	13	20	S3
DGAT1_ins9-11_GGA_S1E1	GGCACCATGAGTTGACGTCG	AAGCTCTTCTCCTACCGCGACGTCAACTCA	10	20	2b,c,d, S5
DGAT1_ins9-11_GGA_S1E2	GGCACCATGAGTTGACGTCG	AAGCTCTTCTCCTATCGCGACGTCAACTCA	10	20	2d, S5
ATP7B-del19-pegRNA1	CAGCACTGTGGTTTCCAAGA	AGAAAGCTGTTCTACTAATCCTCTTGGAAACCACAGTGC	15	24	2k, S9
ATP7B-del19-pegRNA2	CAGCACTGTGGTTTCCAAGA	TCCTAGAAAGCTGTTCTACTAATCCTCTTGGAAACCAC	10	28	2k, S9
ATP7B-del19-pegRNA3	CAGCACTGTGGTTTCCAAGA	TAGAAAGCTGTTCTACTAATCCTCTTGGAAACCACAG	12	25	2k, S9
ABCB11-R1153H-correct-1	AAACAATTCCAATGTTTGAG	CCAGTTCCTCCGCTCAAACATTGGAATTGT	12	18	2g, S7
ABCB11-R1153H-correct-2	CAATTCCAATGTTTGAGTGG	TGTCCAGTTCCTCCGCTCAAACATTGGAAT	12	18	2g, S7
SERPINA1-E342K-correct	TCCCCTCCAGGCCGTGCATA	TCCCTTTCTCGTCGATGGTCAGCACAGCCTTATGCACGGCCTGGAG	12	34	2g, S7

Supplementary Table 2. Sequences of nicking sgRNAs used in prime editing (PE3), HDR, Cas9-cutting, and base editing experiments. Sequence of HDR template used for HDR in *DGAT1* .

nicking sgRNA (PE3)	spacer sequence	Figure
HEK3+90	GTCAACCAGTATCCCGGTGC	1b,c,h, S4
CTNNB1+86/+93	CCACTCATACAGGACTTGGG	1d,e,g,h, S4
ABCB11_482+80	ATGGTGGTAGAGAACAGAAC	S3
DGAT1+46	GGGCTCCCTGCTGGCGCTGA	2b,c,d, S5
DGAT1_PE3b	CCTACCGCGACGTCAACTCA	2d, S5
DGAT1_PE3b+PAMmut	CCTATCGCGACGTCAACTCA	2d, S5
ATP7B_PE3b	AGCTGTTCTACTAATCCTCT	2k, S9
ATP7B-420-39	TCCATGGTGCAAACTACAGA	2k, S9
ABCB11-R1153H+45/+42	CTCTATTGGCAGATGATAGA	2g, S7
SERPINA1-E342K+54	GGGTATGGCCTCTAAAAACA	2g, S7

base edit sgRNA (ABE-NG)	spacer sequence	Figure
ABCB11-R1153H	CTCCACTCAAACATTGGAAT	2g, S7
SERPINA1-E342K	ATCGACAAGAAAGGGACTGA	2g, S7

HDR sgRNA	spacer sequence	Figure
DGAT1_HDR_sgRNA	GGCACCATGAGTTGACGTCG	2f, S6

HDR repair template	HDR template sequence
DGAT1_HDR_template	GCTGTGGAGGCCTGAGTCCACCTCTCCTGCAGTGGGCTCCCTGCTGGCGCTGATGGCGCACACCATCCTCTTCCT CAAGCTCTTCTCCTATCGCGACGTGAATTCATGGTGTCGCAGAGCCAGGGCCAAGGCTGGTGAGGGGCTGCCAGG GGCTGGGGCTGCCTGCTGGGGGGCTGGGCAGCAGCAGGGCCCCAC

Cas9-cutting sgRNA	spacer sequence	Figure
ATP7B_exon3-cas9-KO	CCATGGAATTCCCAGCACTG	S8

Supplementary Table 3. Sequences of PCR and sequencing primers used for amplification and sequencing of target regions. All sequences are shown in 5' to 3' orientation.

PCR and Seq primers	primer sequence	Figure
HEK3_PCR-Fw	ATGTGGGCTGCCTAGAAAGG	1b,c
HEK3_PCR-Rv	GGTGCTGAAAGCCACTGGGC	1b,c
HEK3_Seq-Fw	GGGAAACGCCCATGCAATTAG	1b,c
CTNNB1-ex3-PCR-Fw	AGCAGGAATCTAGTCTGGATGA	1f
CTNNB1-ex3-PCR-Rv	ACTCACTATCCACAGTTCAGCA	1f
CTNNB1-ex3-Seq-Fw	GGATGCAGTACCATTCTTCCAC	1f
ABCB11_482-PCR-Fw	CCACAATGAATTGTTCTGCAGC	S3
ABCB11-482_PCR-Rv	CCTTCTATGACCTCTTAGTTTCTCC	S3
ABCB11-482_Seq-Fw	CCACATTTCAAGAGGAATTGTTTCC	S3
DGAT1_PCR-Fw	ATGCCTGGTTATTGGTGAGC	2b, S6
DGAT1_PCR-Rv	TGAGCTCGTAGCACAAGGTG	2b, S6
DGAT1_Seq-Fw	GCATTCCAGGTTGAGAAGCG	2b, S6
ATP7B_PCR-Fw	CCCCGCATGCAGTAAGTATT	2h, S8, S9
ATP7B_PCR-Rv	AACACAATGCCAGTTATACAAGGA	2i, S7, S8
ATP7B-Seq-Fw	AGGTGAAGTTACGGGGTAGC	2i, S7, S8
ABCB11_1153-PCR-Fw	CTGTAGAGCCAGAGTTCAGG	2g, S7
ABCB11_1153-PCR-Rv	CATTCTACTTCTCCCCATCC	2g, S7
SERPINA1_342-PCR-Fw	GTCAGGAAGATGGACAGAGG	2g, S7
SERPINA1_342-PCR-Rv	GGCAGTTATTTTTGGGTGGG	2g, S7
HEK3_HTS-Fw	ACACTCTTTCCCTACACGACGCTCTTCCGATCTNNNNATGTGGGCTGCCTAGAAAGG	1h
HEK3_HTS-Rv	TGGAGTTCAGACGTGTGCTCTTCCGATCTCCCAGCCAAACTTGTCAACC	1h
CTNNB1_HTS-Fw	ACACTCTTTCCCTACACGACGCTCTTCCGATCTNNNNCAATCTACTAATGCTAATACTGTTTCG	1h
CTNNB1_HTS-Rv	TGGAGTTCAGACGTGTGCTCTTCCGATCTAAGGACTGAGAAAATCCCTGTTC	1h
