# Supplementary material for: Prime editing for functional repair in patient-derived disease models
Source: Nat Commun. 2020 Oct 23;11:5352. doi: 10.1038/s41467-020-19136-7 (PMC7584657; doi:10.1038/s41467-020-19136-7)
Supplement: Supplementary file 3 — Reporting Summary [file 41467_2020_19136_MOESM3_ESM.pdf]

nature research

Corresponding author(s): Sabine A. Fuchs

Last updated by author(s): 25-8-2020

Reporting Summary

Nature Research wishes to improve the reproducibility of the work that we publish. This form provides structure for consistency and transparency in reporting. For further information on Nature Research policies, see our Editorial Policies and the Editorial Policy Checklist.

Statistics

For all statistical analyses, confirm that the following items are present in the figure legend, table legend, main text, or Methods section.

n/a	Confirmed	
☐	☒	The exact sample size (*n*) for each experimental group/condition, given as a discrete number and unit of measurement
☐	☒	A statement on whether measurements were taken from distinct samples or whether the same sample was measured repeatedly
☐	☒	The statistical test(s) used AND whether they are one- or two-sided<br>*Only common tests should be described solely by name; describe more complex techniques in the Methods section.*
☐	☒	A description of all covariates tested
☐	☒	A description of any assumptions or corrections, such as tests of normality and adjustment for multiple comparisons
☐	☒	A full description of the statistical parameters including central tendency (e.g. means) or other basic estimates (e.g. regression coefficient) AND variation (e.g. standard deviation) or associated estimates of uncertainty (e.g. confidence intervals)
☐	☒	For null hypothesis testing, the test statistic (e.g. *F*, *t*, *r*) with confidence intervals, effect sizes, degrees of freedom and *P* value noted<br>*Give P values as exact values whenever suitable.*
☒	☐	For Bayesian analysis, information on the choice of priors and Markov chain Monte Carlo settings
☒	☐	For hierarchical and complex designs, identification of the appropriate level for tests and full reporting of outcomes
☒	☐	Estimates of effect sizes (e.g. Cohen's *d*, Pearson's *r*), indicating how they were calculated

Our web collection on statistics for biologists contains articles on many of the points above.

Software and code

Policy information about availability of computer code

Data collection	For the assessment of organoid reconstitution, organoid growth, and organoid cell death (propidium iodide), fluorescence microscope images were analyzed using an automated organoid counting algorithm written in ImageJ (version 1.51).
Data analysis	For comparisons of multiple groups, an ordinary one-way ANOVA with Holm–Sidak correction for multiple comparisons was used in Prism version 8.4.1 (GraphPad Software, LLC).<br><br>For the analysis of high-throughput amplicon sequencing, alignment of amplicon sequences to reference sequences was performed by Cas-analyzer in HDR mode (Park, J. et al. Bioinformatics 33, 286-288, 2017).<br><br>Whole genome-sequencing reads were mapped against human reference genome hg19 using Burrows-Wheeler Aligner v0.5.924 run in R (version R 4.0). Duplicate sequence reads were marked using Sambamba v0.4.7.32 and realigned per donor using Genome Analysis Toolkit (GATK). We extracted mutational signatures from the whole-genome sequencing data and estimated their contribution to the overall mutational profile as described using an in house developed R package (MutationalPatterns, Blokzijl et al., Genome Med. 10, 33,2018).

For manuscripts utilizing custom algorithms or software that are central to the research but not yet described in published literature, software must be made available to editors and reviewers. We strongly encourage code deposition in a community repository (e.g. GitHub). See the Nature Research guidelines for submitting code & software for further information.

Data

Policy information about availability of data

All manuscripts must include a data availability statement. This statement should provide the following information, where applicable:

- Accession codes, unique identifiers, or web links for publicly available datasets
- A list of figures that have associated raw data
- A description of any restrictions on data availability

Source data for the figures have been provided as Supplementary Table 5. The WGS samples of Fig. 3 and Supplementary Fig. 10 have been submitted to the European Genome-phenome Archive under study number EGAS00001004611.

Field-specific reporting

Please select the one below that is the best fit for your research. If you are not sure, read the appropriate sections before making your selection.

- [x] Life sciences
- [ ] Behavioural & social sciences
- [ ] Ecological, evolutionary & environmental sciences

For a reference copy of the document with all sections, see nature.com/documents/nr-reporting-summary-flat.pdf

Life sciences study design

All studies must disclose on these points even when the disclosure is negative.

Sample size	No statistical methods were used to calculate sample size prior to experiments. In experiments designed to show the ability of prime editors or base editors to alter the genome of primary cells in organoid cultures, sample sizes were chosen to provide a prove of principle in 1-2 experiments with a total of 10-20 clonal organoids. In experiments designed to compare the efficiency of specific prime editing designs or to compare the efficiency of prime editing across different culture types (2D and 3D), sample sizes were chosen to reflect statistical differences in efficiency across 3 repeated experiments or 3 biologically indepedent samples. For genomic and functional repair of DGAT1 organoid lines from two donors with the same mutations were used. For genomic and functional repair of ATP7B, one organoid line from one donor was used for repeated experiments.
Data exclusions	No data was excluded from analysis.
Replication	No attempts at replication of experiments in the paper failed. Data in this paper was reproducible: numbers of experiments (see 'Statistics and reproducibility' section) and source data (see Supplementary Table 5) have been provided.
Randomization	In vitro cultures were paired and distributed into control and experimental prime editing, HDR, or base editing groups for comparison. Specifically, cells from each donor were harvested and pooled into one large pool before each transfection experiment. The different transfection conditions were applied to sub-pools of equal size, taken from this larger pool. Therefore, no bias existed in the allocation of experimental conditions. Unless stated otherwise, clonal organoids grown from GFP+, transfected cells were picked at random before sequencing.
Blinding	For functional assay analyses scored by investigators (counting of growout of organoids in -Rpso1 conditions after CTNNB1 editing and survival of organoids after oleic acid expsore after DGAT1 editing) treatments of organoids were blinded and only unblinded after results had been tabulated. The analysis and quantification of organoids reconstitution and organoid size after transfection with prime-editing plasmids, as well as the quantificaiton of propidium iodide signal after copper exposure, were performed by an automated algortihm written in ImageJ. Therefore, blinding was not relevant for these analyses.

Reporting for specific materials, systems and methods

We require information from authors about some types of materials, experimental systems and methods used in many studies. Here, indicate whether each material, system or method listed is relevant to your study. If you are not sure if a list item applies to your research, read the appropriate section before selecting a response.

Materials & experimental systems

n/a	Involved in the study	
	x	Antibodies
x		Eukaryotic cell lines
x		Palaeontology and archaeology
x		Animals and other organisms
	x	Human research participants
x		Clinical data
x		Dual use research of concern

Methods

n/a	Involved in the study	
x		ChIP-seq
	x	Flow cytometry
x		MRI-based neuroimaging

Antibodies

Antibodies used	Rabbit monoclonal anti-DGAT1; Abcam; 181180<br>Mouse monoclonal anti-B-actin; Santa Cruz Biotechnology; 47778, RRID:AB_626632<br>HRP-conjugated secondary rabbit anti-mouse (1:5000, DAKO p0260)<br>HRP-conjugated secondary swine anti-rabbit(1:5000, DAKO p0217)
Validation	The anti-DGAT1 antibody was validated by lack of western blot staining in intestinal organoids derived from two DGAT1 2x S210del patients, but bright staining in healthy control-derived organoids. No test data was added tot this manuscript for the validation of anti-B-actin antibody. The antibody was used as specified on the datasheet and literature citing the use of this antibody can be found on the companies website: https://www.scbt.com/p/beta-actin-antibody-c4.

Human research participants

Policy information about studies involving human research participants

Population characteristics	Healthy control organoid lines derived from human liver and intestinal biopsies were obtained from anonymous donors (age and gender not disclosed). Biallelic DGAT1 S210del intestinal organoids were derived from two male patients aged 14 and 12 at biopsy. Biallelic ATP7B S430fs (Wilson) liver organoids were derived from an adult male patient aged 28 at biopsy. Biallelic ABCB11 R1153H liver organoids were derived from a female patient aged 15 at biopsy. Biallelic SERPINA1 E342K liver organoids were derived from a male patient aged 7 at biopsy.
Recruitment	Tissue biopsies from liver of a patient with BSEP-deficiency was obtained during a liver transplant procedure in the UMCG, Groningen, and biopsies from duodenum of two patients with DGAT1-deficiency was obtained during diagnostic duodenoscopy in the UMCG, Groningen. Tissue biopsies from livers of patients with Wilson disease and alpha-1 antitrypsin deficiency were obtained during liver transplant procedures in the Erasmus MC, Rotterdam. All biopsies were used after written informed consent. All patient mutations were genetically verified. No self-selection or other biases are applicable given the genetic nature of diseases.
Ethics oversight	The study was approved by the responsible local ethics committees (Institutional Review Board of the University Medical Center Utrecht (STEM: 10-402/K) and Erasmus MC Medical Ethical Committee (MEC-2014-060).

Note that full information on the approval of the study protocol must also be provided in the manuscript.

Flow Cytometry

Plots

Confirm that:

- [x] The axis labels state the marker and fluorochrome used (e.g. CD4-FITC).
- [x] The axis scales are clearly visible. Include numbers along axes only for bottom left plot of group (a 'group' is an analysis of identical markers).
- [x] All plots are contour plots with outliers or pseudocolor plots.
- [x] A numerical value for number of cells or percentage (with statistics) is provided.

Methodology

Sample preparation	2-3 days after electroporation using the GFP plasmid, human intestinal- and liver derived organoid cells were dissociated with TrypLE for 2-3 minutes at 37°C. The cells were washed once using Advanced DMEM/F12 and resuspended in 400 µl FACS buffer (PBS with 2 mM EDTA and 0.5% BSA).
Instrument	FACS Aria, BD Biosciences
Software	FlowJo software was used for data analyses.
Cell population abundance	Typically, 2-10% of the sorted cells were living, GFP+ (transfected) cells. This number differed between different organoid lines and generally decreased after prolonged culturing. Because of this, only organoids of passage <12 were used for transfection experiments.
Gating strategy	SSC/FSC and autofluorescence were used to identify live cells; GFP was used to identify cells transfected with the cocktail of plasmids containing the GFP-reporter plasmid.

- [x] Tick this box to confirm that a figure exemplifying the gating strategy is provided in the Supplementary Information.
