# Supplementary material for: Prime editing for functional repair in patient-derived disease models
Source: Nat Commun. 2020 Oct 23;11:5352. doi: 10.1038/s41467-020-19136-7 (PMC7584657; doi:10.1038/s41467-020-19136-7)
Supplement: Supplementary file 5 — Description of Additional Supplementary Files [file 41467_2020_19136_MOESM5_ESM.pdf]

Title: Supplementary Data 1

Description: In silico predicted off-target sites, SNV, and indel counts for WGS
